# Supplementary material for: Learning curve and surgical outcome of robotic assisted colorectal surgery with ERAS program
Source: Sci Rep. 2022 Nov 29;12:20566. doi: 10.1038/s41598-022-24665-w (PMC9709162; doi:10.1038/s41598-022-24665-w)
Supplement: Supplementary file 2 — Supplementary Information 2. [file 41598_2022_24665_MOESM2_ESM.zip › RAL Raw data-Ñ[▒K/094/Patho_ñw╝╨░O▒Kñσ.pdf]

臺中榮民總醫院 臺中市臺灣大道四段1650號 病理部活體組織切片 臨床醫學實驗及病理診斷中心 檢驗(查)報告

[redacted]

Pathologic diagnosis:
1. Rectum, robotic low anterior resection --- a. Status post polypectomy with tatoo and focal foreign body type giant cell reaction. No residual tumor is seen. The bilateral cut ends and perirectal lymph nodes (0/16) are free of tumor. b. Hyperplastic polyp.
2. Rectum, cut end, distal, resection --- Unremarkable.
3. IMA root, dissection --- Unremarkable fibroadipose tissue, nerve bundles and lymph nodes (0/5).

Ancillary study for diagnosis:
Step cut x 3 done for section C; recut done for section D and E.

Prognostic and predictive factor:
(Combined with report of polypectomy: S1009984)
1. Tumor size: 1.1 cm.
2. Depth of invasion: Submucosa.
3. Histologic grade: G2: Moderately differentiated.
4. Angiolymphatic invasion: Not identified.
5. Perineural invasion: Not identified.
6. Tumor deposits (discontinuous extramural extension): Not identified.
7. Macroscopic tumor perforation: Not identified.
8. Surgical Margins:
a) Proximal Margin: Uninvolved by invasive carcinoma, distance of scar from margin: 11 cm.
b) Distal Margin: Uninvolved by invasive carcinoma, distance of scar from margin: 1 cm.
c) Radial or Mesenteric Margin: Uninvolved by invasive carcinoma.
9. Lymph node status: Uninvolved (0/21).
10. Extranodal involvement: Not identified.
11. Tumor regression grading S/P CCRT: No known presurgical therapy.
12. Type of polyp in which invasive carcinoma arose: Tubular adenoma.
13. Additional pathologic findings: Hyperplastic polyp.
14. Pathological TNM Stage: pT1N0(According to the eighth edition, American Joint Committee on Cancer Staging Guidelines for Tumors).
15. TNM descriptors: Not applicable.

Gross description:
The specimen consists of 1) a segment of colon, 12.5 cm in length and 4.5 cm in proximal cut end circumference, 5.5 cm in distal circumference, and perirectal soft tissue. A scar, 0.5x0.5 cm, marked by surgical tie is noted on the mucosa, 1 cm from the distal cut end. On cross section, there is no tumor seen grossly. The both cut ends are unremarkable. 2) distal cut end, a piece of tan mucosa-covered soft tissue, 3x2x1.1 cm. 3) IMA root, 2 pieces of tan yellow soft tissue, up to 3.5x2.5x1 cm. Representative part for section: A) proximal cut end B) distal cut end C-E) all of scar F) radial margin G-H) specimen 2-3 J-L) lymph nodes. Blue ink indicated serosa; yellow ink indicated radial margin.
#T-59600_2 #M-62510_2 0 1105 SP0000
#T-C4560_2 #M-09183_2 0 1105 000000

已電子簽章

敬告：本單僅供醫師參考使用，不具任何申請效力！！
請病患妥為保管，若不慎遺失需自負個人資料外洩責任！
